# Indocyanine Green—Mediated Photodynamic Therapy Reduces Methicillin-Resistant *Staphylococcus aureus* Drug Resistance

**DOI:** 10.3390/jcm8030411

**Published:** 2019-03-25

**Authors:** Tak-Wah Wong, Shu-Zhen Liao, Wen-Chien Ko, Chi-Jung Wu, Shin Bei Wu, Yin-Ching Chuang, I-Hsiu Huang

**Affiliations:** 1 Department of Dermatology, National Cheng Kung University Hospital, Department of Biochemistry and Molecular Biology, College of Medicine, National Cheng Kung University, Tainan 704, Taiwan; mymini0902@hotmail.com (S.-Z.L.); snobbish1522@gmail.com (S.B.W.); 2 Center of Applied Nanomedicine, National Cheng Kung University, Tainan 70101, Taiwan; 3 Division of Infectious Diseases, Department of Medicine, National Cheng Kung University Hospital, College of Medicine, National Cheng Kung University, Tainan 704, Taiwan; winston@mail.ncku.edu.tw (W.-C.K.); wu.chijung@msa.hinet.net (C.-J.W.); 4 National Institutes of Infectious Diseases and Vaccinology, National Health Research Institutes, Tainan 70456, Taiwan; 5 Department of Internal Medicine, Chi Mei Medical Center, Liouying, Tainan 72263, Taiwan; chuangkenneth@hotmail.com; 6 Department of Medical Research, Chi Mei Medical Center, Tainan 72263, Taiwan; 7 Department of Microbiology and Immunology, College of Medicine, National Cheng Kung University, Tainan 70101, Taiwan

**Keywords:** drug resistance, indocyanine green, MRSA, photodynamic, photothermal, wound infection

## Abstract

Methicillin-resistant *Staphylococcus aureus* (MRSA) skin-wound infections are associated with considerable morbidity and mortality. Indocyanine green (ICG), a safe and inexpensive dye used in clinical imaging, can be activated by near-infrared in photodynamic therapy (PDT) and photothermal therapy (PTT) to effectively kill MRSA. However, how this treatment affects MRSA drug sensitivity remains unknown. The drug-sensitivity phenotypes, bacterial growth rate, and cell-wall thickness of three MRSA strains were analyzed after ICG-PDT. Drug-resistant gene expressions were determined by polymerase chain reaction (PCR) and quantitative reverse transcription (qRT)-PCR. Related protein expressions were examined with immunoblotting. Drug sensitivity was further evaluated in animal models. MRSA that survived the treatment grew faster, and the cell wall became thinner compared to parental cells. These cells became more sensitive to oxacillin, which was partly related to mecA complex gene deletion. Skin necrosis caused by ICG-PDT-treated MRSA infection was smaller and healed faster than that infected with parental cells. With oxacillin therapy, no bacteria could be isolated from mouse lung tissue infected with ICG-PDT-treated MRSA. ICG-PDT drives MRSA toward an oxacillin-sensitive phenotype. It has the potential to develop into an alternative or adjuvant clinical treatment against MRSA wound infections.

## 1. Introduction

*Staphylococcus aureus* is a gram-positive normal resident flora of the human skin and respiratory tract that causes a wide range of clinical infections [1,2]. In the United States, the annual incidence of *S. aureus* bacteremia is 4–38 per 100,000 person years. The 30-day all-cause mortality of *S. aureus* bacteremia is 20% [3,4]. In the 1940s, *S. aureus* was sensitive to penicillin, a β-lactam antibiotic that inhibits the formation of peptidoglycan crosslinks in the bacterial cell wall and leads to cell death [5]. The first methicillin-resistant *S. aureus* (MRSA) was isolated in the UK in 1961 [6]. To date, MRSA infections have become one of the most concerning global health issues because of the high mortality, long hospital stays, and high healthcare costs.

The well-documented mechanism of drug resistance in MRSA is mediated by acquisition of the *mecA* gene, which translates into a novel penicillin-binding protein (PBP), PBP2a [7]. PBP2a decreases MRSA’s affinity for methicillin and thus allows MRSA to survive methicillin treatments. This resistant phenotype is regulated by MecR1 and MecI [8]. MRSA can also acquire other genes that induce resistance to other antibiotics such as tetracycline and linezolid. Currently, the drug of choice to treat MRSA infection is limited [9]. Vancomycin, a glycopeptide antibiotic, has become the last resort against MRSA infection. However, vancomycin resistance has also become a new challenge in recent years [10,11]. New and novel therapies are urgently needed for the treatment and prevention of drug-resistant *S. aureus* infections.

Antimicrobial photodynamic therapy (aPDT) induces cell death by generating lethal oxidative stress. When a photosensitizer (PS) is activated by a specific wavelength of light, oxidative stress is generated via Type I and II reactions. Type I reaction generates reactive oxygen species including superoxide anion (O_2_^•−^), hydroxyl radicals (OH^•^), H_2_O_2_, and carbonate radical anions (CO_3_^•−^) via electron transfers from the excited PS to molecular oxygen inside a cell or in the microenvironment. Singlet oxygen is produced in a Type II reaction through direct energy transfer from the excited PS to molecular oxygen. Both reactions are believed to participate in aPDT, and the ratio of the two mechanisms is unique for each PS [12,13]. aPDT has become an attractive alternative to antibiotics [14,15,16] in recent decades because of its potential to effectively kill multidrug-resistant bacteria and its low tendency to induce drug resistance [13]. aPDT affects multiple cellular functions, including DNA interaction [17], membrane integrity [18], protease activity [19,20], and lipopolysaccharide (LPS) bioactivity within a bacterium [20]. Unlike aPDT, an antibiotic usually interrupts only one function within cells. This explains the difficulty of a bacterium to develop resistance to aPDT. However, only three PSs (methylene blue, toluidine blue, and indocyanine green) have been approved in clinical use to date [13].

Indocyanine green (ICG) has been used as a safe and inexpensive fluorescence imaging dye in hepatic, cardiac, and ophthalmologic perfusion examinations since the mid-1950s [21,22,23,24]. The benefits of using ICG as a PS and photothermal agent include a high safety profile and a relatively low cost compared to other PSs [25,26]. Its near-infrared (NIR) (600–950 nm) excitation wavelength that can penetrate deeper within the tissue is another advantage. Some reports say that ICG is a photothermal agent rather than a PS due to its poor singlet oxygen yield and relatively high heat generation during irradiation (photothermal therapy, PTT) [27,28] Nevertheless, the hyperthermic effects of ICG after photoactivation may be beneficial to a patient with a malignant tumor. In 1998, Abels et al. made the first clinical report of using ICG as a PS to treat 16 AIDS-associated cutaneous Kaposi’s sarcomas with complete remission after one treatment [29].

Recently, ICG-mediated PDT/PTT has been reported in antimicrobial research [30,31,32]. Omar et al. reported a 6–7 log reduction of viable *S. aureus* and *S. pyogenes* isolates from wounds using ICG-PDT/PTT [33]. Topaloglu et al. used a diode laser to activate ICG with almost 100% MRSA growth inhibition [34]. We have shown that MRSA pretreated with 0.1% H_2_O_2_ before ICG-PDT reduced MRSA growth by seven logs with a light intensity that was harmless to human fibroblasts [26]. The photothermal effects of ICG in our system were excluded by maintaining the temperature at 37 °C. It was achieved by cooling with electric fans during irradiation [26]. Therefore, ICG-photodynamic rather than photothermal inactivation of MRSA is the predominant effect in the present study.

Most studies nowadays focus on how to enhance the bactericidal effects of ICG-PDT/PTT on MRSA. Little is known about the physiology of the bacteria that survive the treatment. In this study, we investigated how MRSA responds to antibiotics after ICG-PDT. We found that ICG-PDT-treated MRSA became more sensitive to oxacillin, and the susceptible phenotype persisted for more than 10 passages. The results suggest that ICG-PDT has great potential in eliminating MRSA.

## 2. Materials and Methods

### 2.1. Bacterial Isolates

Four methicillin-sensitive strains, MSSA/JD038, MSSA/31987, MSSA/31784, and MSSA/31596, and two MRSA strains, MRSA/JD004 and MRSA/JD39452, were isolated from patients’ wounds in the National Cheng Kung University Hospital, Taiwan. The other MRSA strain (ATCC^®^ BAA-1556^™^) was purchased from the American Type Culture Collection (VA, USA). All bacteria were stored at −80 °C in Microbank™ (Bio TRADING, Mijdrecht, the Netherlands) until used. The bacteria were grown in Luria-Bertani (LB) broth or on agar plates at 37 °C, and Mueller–Hinton (MH) agar for a drug-susceptibility test. Two colonies from each strain on the agar plates were used in all experiments, and experiments were repeated at least three times to achieve unbiased results. To minimize the amount of animal used, in vivo experiments were repeated twice.

### 2.2. Photodynamic Therapy System and ICG Photoactivation

The system was composed of a mirror at the bottom and surrounded by aluminum foil to reflect light. An NIR lamp was hung over the center of the mirror. The irradiation system was maintained at 37 ± 1 °C by electric fans installed on both sides [26]. A 96-well culture plate containing bacteria and ICG was placed at the center during irradiation.

ICG (Diagnogreen, Daiichi Sankyo Taiwan, Taipei, Taiwan) was prepared freshly by dissolving in sterile water according to the manufacturer’s instructions. Different concentrations of ICG (50 µL) and 50 µL of a bacterial suspension (1 × 10^7^ CFU/mL) were placed into a well of a 96-well plate in triplicate for each condition. The plate was exposed to NIR light with 100 J/cm^2^ at 65.5 mW/cm^2^ after 10 min of incubation at 37 °C. This condition was selected to achieve MRSA growth inhibition by 0.5–2 log (68–99% inhibition) to mimic the clinical scenario in which many bacteria may survive after a low dose of PDT treatment. A higher inhibitory effect can be achieved with other conditions [26]. Four experimental groups were included: absolute control (without light and ICG), dark control (exposed to ICG and kept in the dark), light control (exposed to light without ICG), and PDT (exposed to light with ICG). Bacteria counts in CFU/mL were calculated using serial dilution and bacterial drop-plate count methods [35]. In brief, an agar plate was allocated into 4 quadrants; each quadrant was reserved for one dilution in the 10-fold serial dilutions. Each quadrant contained 3 drops of 20 µL bacterial suspension. Results are based on 3 separate experiments in triplicates.

### 2.3. Growth Curves

To evaluate the influence of ICG-PDT on bacterial growth, growth curves of different MRSA strains before and after exposure to PDT were compared to MSSA. The growth dynamics of different bacteria were studied by plotting cell growth (represented by absorbance values at 600 nm) versus incubation time.

### 2.4. Antibiotic Susceptibility Testing

The disk-diffusion method and minimum-inhibitory-concentration (MIC) determination were used to measure antimicrobial susceptibility [26]. According to the guidelines of the Clinical and Laboratory Standards Institute (CLSI, Table S1), disks (Oxoid^TM^ Disc, Thermo Fisher Scientific Inc., Waltham, MA, USA) containing 1 µg oxacillin (OX), 30 µg cefoxitin (FOX), 30 µg vancomycin (VA), 5 µg rifampin (RA), 30 µg tetracycline (TE), 15 µg erythromycin (E), and 300 units polymyxin B (PB) were placed on an MH agar plate prespread with bacteria with a McFarland turbidity of 0.5 (1 × 10^8^ CFU/mL MRSA, collected from different treatments as described above). The diameter of each inhibition zone was measured after 18 h of incubation at 37 °C. The larger the diameter, the more sensitive the bacteria are to the antibiotic in the disk.

For MIC measurement, the bacteria were resuspended in normal saline at a concentration of 5 × 10^5^ CFU/mL and exposed to 2-fold diluted concentrations of cefoxitin (range: 512–1 mg/L) and oxacillin (range: 32–0.0625 mg/L) in a 96-well plate incubated at 37 °C for 24 h.

### 2.5. Transmission Electron Microscopy

Transmission electron microscopy (TEM) was used to measure cell-wall thickness. Bacteria after ICG-PDT (1 × 10^7^ CFU/mL) were pelleted by centrifuging and resuspended with 2.5% glutaraldehyde at 4 °C for 30 min. The fixed samples were sent to the core lab of the National Cheng Kung University Hospital for further processing. Cell-wall thickness was determined using photographs taken at a final TEM magnification of 150,000× (Hitachi H7650, Tokyo, Japan) at 60 kV. Five bacteria of each MRSA strain were randomly chosen and blinded to the observer. The cell-wall thickness of each bacterium was measured at more than 5 sites.

### 2.6. Polymerase Chain Reaction

Cell suspensions after different treatments were centrifuged into a pellet and resuspended in 0.8 mg/mL proteinase K and 40 mg/mL lysozyme at 37 °C for 1 h. DNA was extracted with a DNA extraction kit (Zymo Research, Irvine, CA, USA) following the manufacturer’s manual. DNA quantity and quality were determined by an OD260/280 ratio with NanoDrop™ 2000 (Thermo Scientific, Waltham, MA, USA). Polymerase chain reaction (PCR) reaction mixtures contained 50 ng of chromosomal DNA, oligonucleotide primers (Table S2 [36,37]; 0.2 µM), master mix (Takara Bio Inc., Kyoto, Japan) containing hot-start PCR enzyme, optimized buffer, dNTP mixture, gel loading dye, and a density reagent in a final volume of 25 µL. A PCR Thermal Cycler (Applied Biosystems 2720, Foster City, CA, USA) was used for amplification with an initial denaturation step (94 °C, 5 min); 30 cycles of denaturation (94 °C, 5 s), annealing (depending on primer pairs, 10 s), extension (72 °C, 25 s); and a final elongation at 72 °C for 3 min. PCR products were electrophoresed in a 1.5% agarose gel containing 1 µL/10 mL nucleic acid stain (HealthView^TM^, Genomics BioSci, Taipei, Taiwan) in a TBE buffer at 100 V for 30 min and visualized with a UV transilluminator.

### 2.7. Quantitative Reverse Transcription Polymerase Chain Reaction

*mecA* gene expression was quantitated with reverse-transcription PCR (RT-PCR). Bacteria were grown in LB broth after different treatments. Cell suspension was centrifuged into a pellet and resuspended in 1 mL of RNAprotect Bacteria Reagent (QIAGEN, Germantown, MD, USA) at room temperature for 5 min to stabilize the RNA. RNA was extracted with an RNA extraction kit (QIAGEN, Germantown, MD, USA) following the manufacturer’s instruction. RNA quantity and quality were determined using NanoDrop™ 2000 (Thermo Scientific, Waltham, MA, USA). Quantitative RT-PCR (qRT-PCR) was performed using a 2-step reaction. RNA was converted to cDNA using reverse transcriptase (Invitrogen, Carlsbad, CA, USA). Real-time PCR reaction mixtures contained 45 ng cDNA, oligonucleotide primers (0.5 µM), and master mix (Fast SYBR Green Master Mix, Applied Biosystems, Foster City, CA, USA), to a final volume of 20 µL. A real-time thermal cycler (Applied Biosystems StepOne™, Foster City, CA, USA) was used for amplification with an initial denaturation at 95 °C for 20 s; 40 cycles of denaturation (95 °C, 3 s), and annealing (60 °C, 30 s). Nonspecific bindings were analyzed with the melting curve. Data were calculated with Ct value using StepOne™ Software (version 2.2.2, Applied Biosystems, Foster City, CA, USA).

### 2.8. Western Blot

Bacteria collected from different treatments were grown to the log phase, disrupted using a sonicator (BD, Franklin Lakes, NJ, USA), and precipitated with trichloroacetic acid and acetone. Protein concentration was detected with a BCA protein assay reagent kit (Pierce, Rockford, IL, USA). Then, 25 µg protein samples were separated on 10% SDS-polyacrylamide gel and electrophoresed at 100 V for 80 min. Samples were transferred to a polyvinylidene difluoride (PVDF) membrane (Bio-rad, Hercules, CA, USA) with 350 mA for 90 min. The membrane was probed with an antibody against PBP2a (RayBiotech, Norcross, GA, USA). Anti-GAPDH (Pierce, Rockford, IL, USA) was used to ascertain equal loading. Horseradish peroxidase (HRP)-conjugated antibody (Jackson immunoresearch, West Grove, PA, USA) and the enhanced chemiluminescence (GE Healthcare, Buckinghamshire, UK) were used for evaluation.

### 2.9. In Vivo

#### 2.9.1. Murine Skin Infection Model

Six-to-eight-week-old male C57BL/6 mice were purchased from the Laboratory Animal Service Center, National Cheng Kung University. Animals were kept in a temperature-controlled room with a 12-hour day and light cycle, with food and water ad libitum. Animal breeding and experimental procedures were performed after approval from the review committee of the Laboratory Animal Service Center of the university. Mice were divided into 2 groups (6 in each group), either infected with MRSA/JD004 or MRSA/JD004-T1 (bacteria that survived after one ICG-PDT treatment). Fifty microliters of a bacterial suspension (1.5–1.9 × 10^7^ CFU/mL) were injected subcutaneously into the left upper back of each mouse after anesthesia with intraperitoneal injection of a 5-fold diluted solution of a combination of 25 mg/mL tiletamine, 25 mg/mL zolazepam (0.005 mL/g body weight; Zoletil 50^TM^, Virbac, Carros, France), and xylazine (0.08 mg/g body weight; Virbac, Carros, France). Oxacillin was injected intraperitoneally at a dose of 400 mg/kg, 15 min after bacterial inoculation. Necrotic skin sites were recorded with a digital camera until the wounds completely healed. The area of skin necrosis was calculated using the formula *l* × *w*, where *l* is length and *w* is width [38]. To assess the bacterial count in the skin lesions, 5 more mice from each group were sacrificed 3 days after subcutaneous infection. The necrotic skin was excised, weighed, and homogenized in the Mini Beadbeater-24 (Biospec Products, Bartlesville, OK, USA) with 3450 strokes/min in 1 g glass beads (2.7 mm, Biospec Products, Bartlesville, OK, USA) and 500 µL PBS for 1 min. The homogenized tissue lysate was serially diluted in PBS and plated on agar. The colonies were enumerated 20 h after incubation at 37 °C.

#### 2.9.2. Murine Pneumonia Model

Eight-to-eleven-week-old male C57BL/6 mice were divided into 2 groups (5 in each group), either infected with MRSA/JD004 or MRSA/JD004-T1. After anesthesia, 10 µL of a MRSA suspension (1.2–1.6 × 10^8^ CFU/mL) were inoculated into the nose of a mouse [38]. Fifteen minutes after infection, the first dose of oxacillin (400 mg/kg) was injected intraperitoneally, followed by 2 doses at the second and third hour, respectively. Mice were sacrificed 24 h after inoculation to quantify bacterial burden in the lung. The left lung was homogenized in 1 mL PBS. After homogenization, the mixture was serially diluted in PBS and plated on an LB agar. Colonies were enumerated 16–18 h after incubation at 37 °C.

### 2.10. Statistics

Statistical significance was analyzed with GraphPad Prism software (Version 5.0, GraphPad Software, Inc., San Diego, CA, USA). One-way analyses of variance (ANOVA) were performed to assess differences between the experiments of more than 2 subgroups. Two-way ANOVA was used to examine the influences of two different independent variables. Bonferroni correction was used for the effect of multiple comparisons. Two separate animal experiments were performed. In vitro experiments for each condition were repeated at least 3 times. A *p*-value < 0.05 was considered statistically significant.

## 3. Results

### 3.1. Photodynamic Inactivation on Three MRSA Strains

To mimic the clinical scenario in which many MRSA may survive after a low dose of ICG-PDT, three MRSA isolates were subjected to less bactericidal ICG-PDT treatment. The presence of 25 µg/mL of ICG and exposure to an NIR lamp with 100 J/cm^2^ at 65.5 mW/cm^2^ inhibited 2 log cell growth of MRSA/JD004 (*** *p* < 0.001, Bonferroni *t*-method, Figure S1). The same ICG-PDT treatment inhibited 1 log and 0.5 log cell growth in BAA-1556 and MRSA/JD39452, respectively (** *p* < 0.01, Bonferroni, Figure S1).

### 3.2. mec Complex Typing of MRSA Strains

In order to characterize drug-sensitivity gene alteration in these three strains, multiplex PCR was performed for *mec* gene-complex genotyping. MRSA/JD004 and MRSA/JD39452 belonged to Class A, while BAA-1556 belonged to Class B (Figure S2) [39].

### 3.3. Physiological MRSA Changes after ICG-PDT

**Figure 1:**
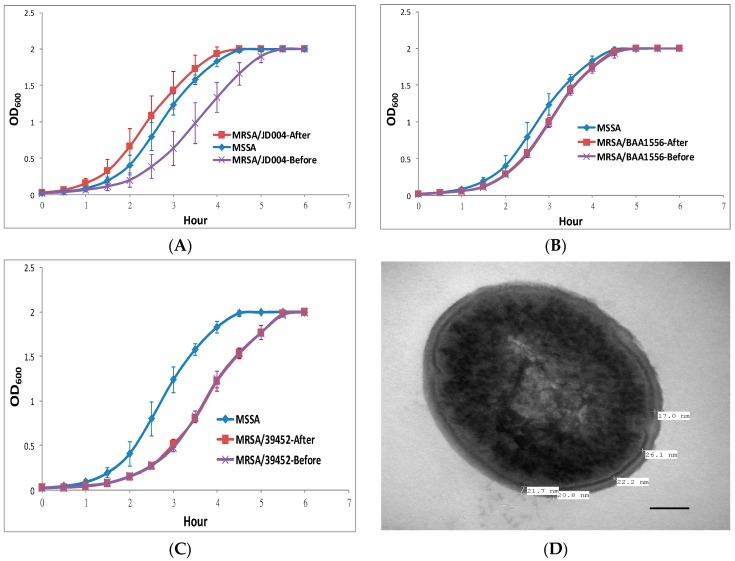

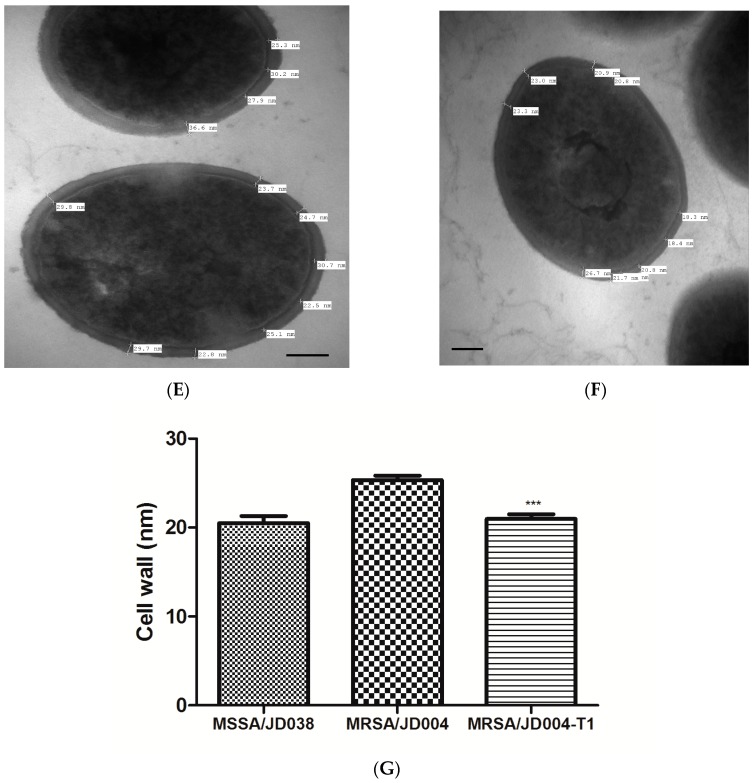
Changes in methicillin-resistant *S. aureus* (MRSA) growth rate and cell-wall thickness after indocyanine green photodynamic therapy (ICG-PDT). (**A**) MRSA/JD004, (**B**) MRSA/BAA-1556, and (**C**) MRSA/39452 exposed to near-infrared 100 J/cm^2^ at 65.5 mW/cm^2^ in the presence of 25 µg/mL of ICG. MRSA/JD004 grew faster after ICG-PDT. Growth rate was similar to the MSSA strain (*** *p* < 0.001, Bonferroni t-method). Methicillin-sensitive *S. aureus* (MSSA) growth curve was averaged and pooled from four different MSSA strains in three separate experiments in duplicates, while MRSA growth curves were pooled from three separate experiments in duplicate of each strain. MRSA/BAA-1556 and MRSA/39452 growth curves remained the same after treatment. The changes in MRSA/JD004 cell-wall thickness were measured under transmission electron microscopy (TEM) after ICG-PDT. For each bacterium, at least five measurements of cell-wall thickness were taken. (**D**) MSSA/JD038, (**E**) MRSA/JD004, (**F**) MRSA/JD004-T1: MRSA/JD004 after one ICG-PDT treatment. (**G)** Average data pooled from five bacteria in each condition (*** *p* < 0.001, Bonferroni *t* method). Bar = 100 nm.

In order to determine whether ICG-PDT affected bacterial growth, the growth curves of MRSA isolates were obtained before and after treatment and compared with four MSSA strains. As shown in Figure 1, all three MRSA strains grew slower in comparison to MSSA before ICG-PDT. After treatment, MRSA/JD004 grew faster and in a similar pattern to MSSA (***p < 0.001; before and after ICG-PDT/PTT; Bonferroni *t*-method, Figure 1A). On the other hand, the growth curves of BAA-1556 and MRSA/JD39452 were not significantly different after ICG-PDT (*p* > 0.05).

Cell-wall thickness is one of the critical factors in drug resistance [40,41,42]. Cell-wall thickness was measured using TEM after ICG-PDT. The average thickness of the MRSA/JD004 cell wall was reduced from 25.3 ± 1 to 20.9 ± 1 nm (17.4% reduction, *** *p* < 0.001, Bonferroni *t*-method), which was similar to MSSA cell-wall thickness (Figure 1G).

### 3.4. Antimicrobial Susceptibility Phenotype Altered by ICG-PDT

**Figure 2:**
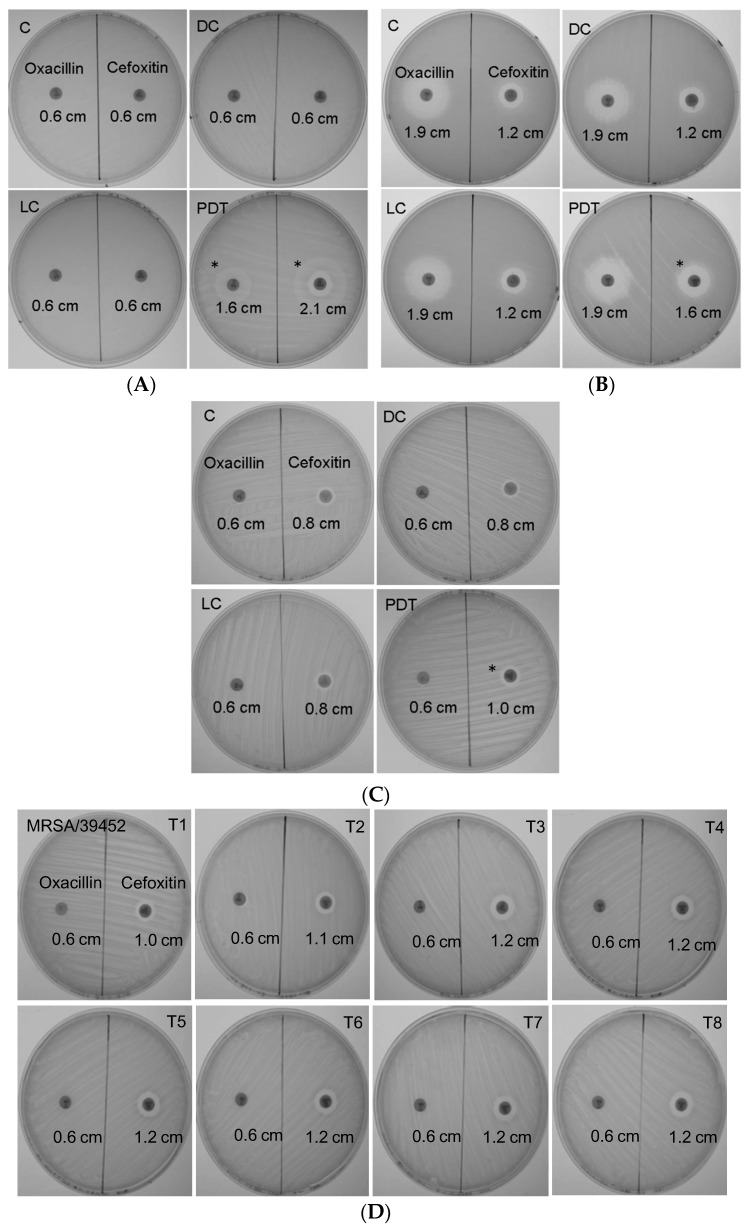
Alterations in MRSA drug sensitivity after ICG-PDT. (**A**) MRSA/JD004, (**B**) MRSA/BAA-1556, and (**C**) MRSA/39452 were incubated with 25 µg/mL of ICG and exposed to 100 J/cm^2^ near-infrared at 65.5 mW/cm^2^; (**D**) MRSA/39452 was treated repeatedly with ICG-PDT up to eight times. The clear zones were created by MRSA growth inhibition by antibiotic in the disks. (**A**) In MRSA/JD004, the oxacillin inhibition zone increased from 0.6 to 1.6 cm after PDT (166.7% increment), and the cefoxitin inhibition zone increased from 0.6 to 2.1 cm (250% increment). (**B**) The oxacillin clear zone did not change in MRSA/BAA-1556, but the cefoxitin clear zone increased from 1.2 to 1.6 cm (33% increment, Figure 2B) after PDT. (**C**) In MRSA/39452, there was a small increase in the cefoxitin inhibition zone (0.8 to 1.0 cm; 25% increment) and no change in the oxacillin inhibition zone. (**D**) In MRSA/39452, the oxacillin diameter did not change after two more repeated treatments, yet the cefoxitin inhibition zone further increased by 20%. The changes of the drug-sensitivity phenotype were maintained until the eighth repeated PDT. T1–T8: number indicates the number of times exposed to PDT. MRSA that survived from the previous PDT treatment were used for the subsequent treatment. The above are representative data of three separate experiments. C: absolute control; DC: dark control; LC: light control; PDT: photodynamic therapy. * sensitive to oxacillin.

To determine whether antibiotic susceptibility was changed in MRSA that survived after ICG-PDT, zone-of-inhibition experiments were performed. Figure 2 shows representative data from three separate experiments. The oxacillin disk inhibition-zone diameters did not change in all tested MRSA strains except for MRSA/JD004, where they increased from 0.6 to 1.6 cm (166.7% increment, Figure 2A) after treatment. Interestingly, the diameter of cefoxitin’s clear inhibition zone increased in all strains. The cefoxitin disk inhibitory-zone diameter of MRSA/JD004 increased from 0.6 to 2.1 cm (250% increment, PDT, Figure 2A); from 1.2 to 1.6 cm in BAA-1556 (33% increment, Figure 2B); and from 0.8 to 1.0 cm in MRSA/JD39452 (25% increment, Figure 2C). We further examined whether repeated treatments would change MRSA drug sensitivity to antibiotics. Interestingly, repeating ICG-PDT up to eight times did not affect oxacillin sensitivity, but enhanced cefoxitin sensitivity (1.0 to 1.2 cm, 20% increment in diameter) in MRSA/JD38452, the most ICG-PDT-resistant strain.

Drug susceptibility was further confirmed using MIC assay. The oxacillin MIC of MRSA/JD004 declined from >32 to 2 mg/L, compatible with drug-sensitive criteria according to CLSI guidelines (Table S1). BAA-1556 and MRSA/JD39452 showed no change in MIC (>32 mg/L).

**Figure 3:**
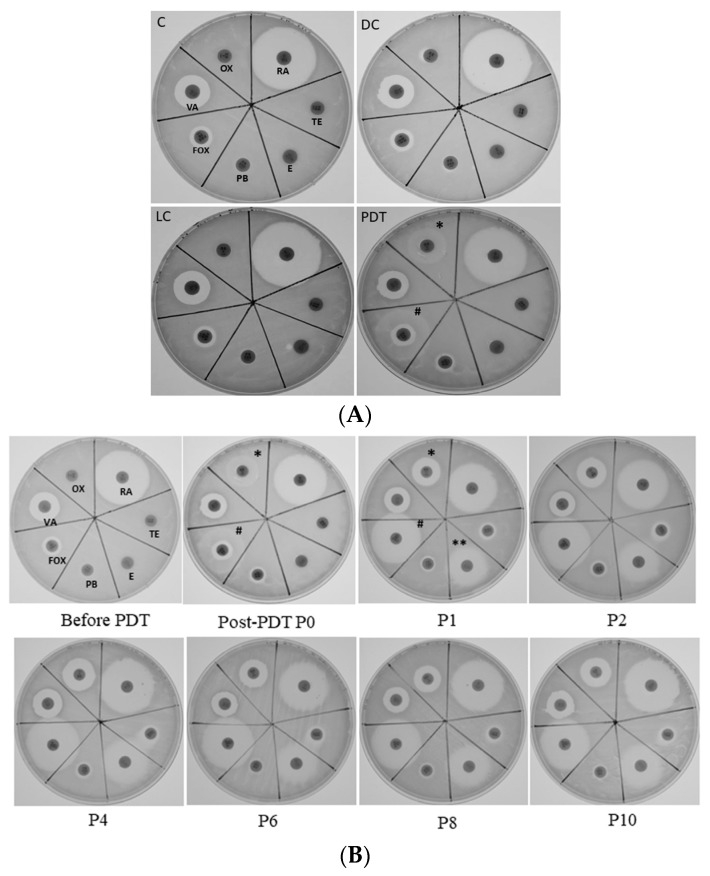
MRSA/JD004 susceptibility to different antibiotics after ICG-PDT treatment. MRSA/JD004 were incubated with 25 µg/mL of ICG and exposed to 100 J/cm^2^ near infrared at 65.5 mW/cm^2^. (**A**) Clear zones demonstrated MRSA became sensitive to oxacillin (*OX) and cefoxitin (#FOX) after PDT treatment. (**B**) Sensitive phenotype persisted up to 10 passages (P10). Note that bacteria become sensitive to erythromycin (**E) in passage 1 (P1) cells and persisted thereafter. VA: vancomycin; OX: oxacillin; RA: rifampin; TE: tetracycline; E: erythromycin; PB: polymyxin B; FOX: cefoxitin.

To further clarify whether ICG-PDT affected MRSA susceptibility to antibiotics by means other than altering cell-wall thickness, MRSA/JD004 was cultured with disks containing different antibiotics. As shown in Figure 3, bacteria become more sensitive to oxacillin (* in Figure 3A) and cefoxitin (# in Figure 3A) after one ICG-PDT treatment. Interestingly, after one passage of the cells, they became sensitive to erythromycin (** in P1, Figure 3B), and the phenotypes persisted for at least 10 passages. A slight increase of the inhibition-zone diameter was also noted in tetracycline, which persisted for 10 passages (Figure 3B).

### 3.5. Gene Alterations after ICG-PDT

**Figure 4:**
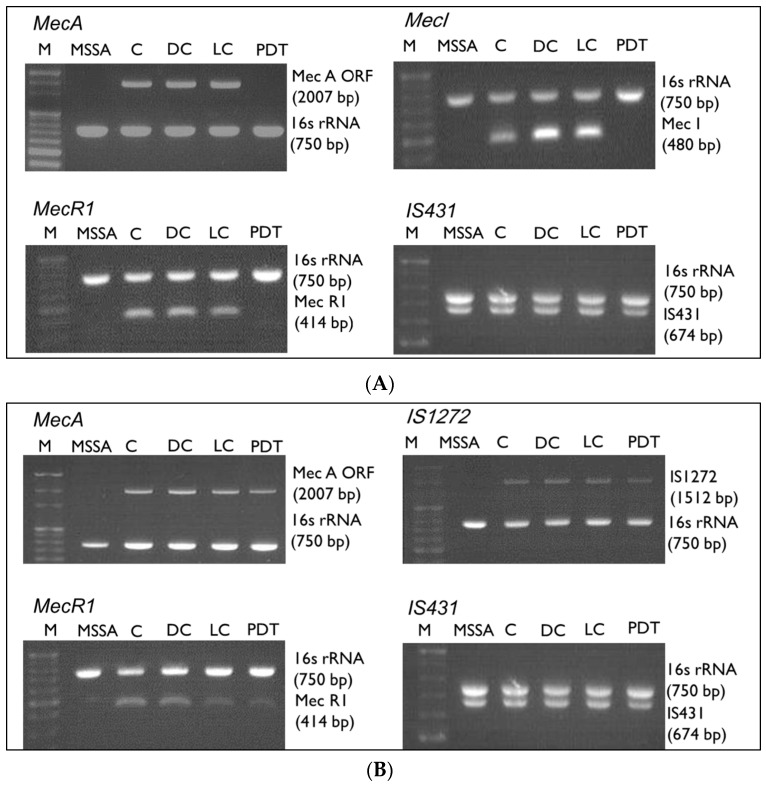

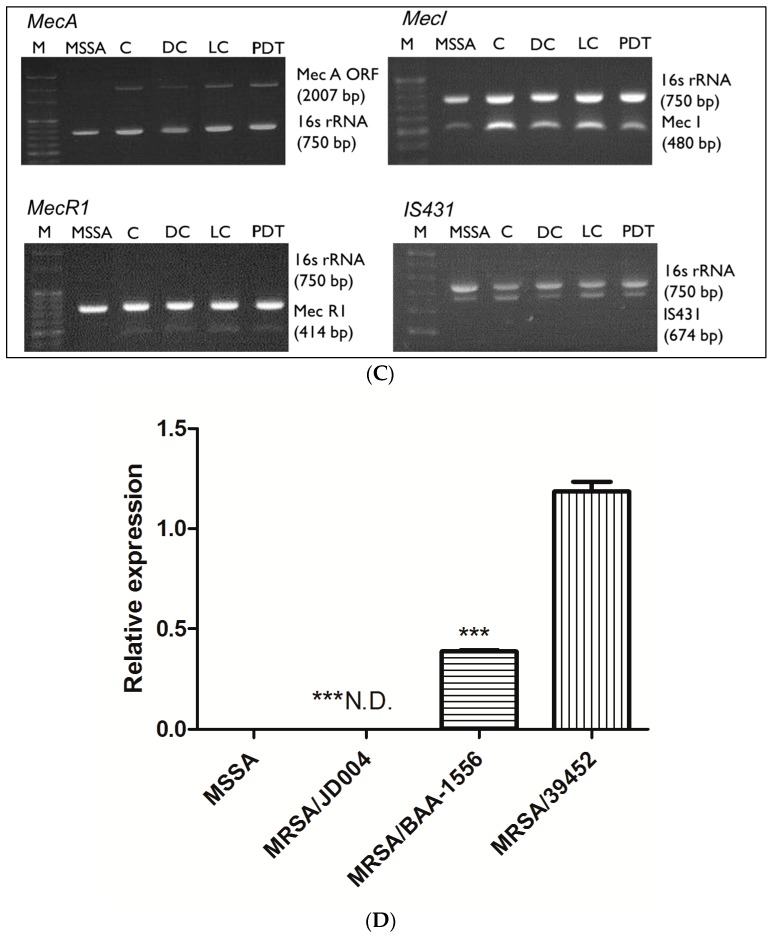
*mecA* complex after ICG-PDT in MRSA/JD004, MRSA/BAA-1556, and MRSA/39452. Presence of *mecA*, *mecI*, *mecR1*, *IS431*, and *IS1272* genes was examined after treatment. (**A**) After PDT, the *mecA*, *mecI*, and *mecR1* genes were undetected in MRSA/JD004 except for *IS431*. (**B**,**C**) There are no changes in the *mecA* complex after treatment in MRSA/BAA-1556 and MRSA/39452. Lane 1: marker; lane 2: MSSA; lane 3: absolute control; lane 4: DC (dark control); lane 5: LC (light control); lane 6: PDT treatment. (**D**) Quantitative changes of *mecA* mRNA within three clinical MRSA isolates after treatment. *mecA* mRNA was analyzed by qRT-PCR. No *mecA* was detected in MSSA and MRSA/JD004 after PDT. *mecA* expression decreased about twofold in MRSA/BAA-1556 after PDT. No significant changes in *mecA* level in MRSA/39452. Results shown in (D) are the mean of three independent determinations ± standard deviation. *** *p* < 0.001, Bonferroni *t* method.

To determine whether antibiotic-resistance genes were altered by ICG-PDT, PCR was performed to identify the presence of the *mec* gene complex in the tested strains (Figure 4). All *mec* complexes except for *IS431* were deleted in MRSA/JD004 (Figure 4A) after ICG-PDT. No alteration was observed in BAA-1556 (Figure 4B) and MRSA/JD39452 (Figure 4C). These changes were further quantified by qRT-PCR (Figure 4D) of the *mecA* gene. After ICG-PDT, no *mecA* expression was detected in MRSA/JD004. Interestingly, *mecA* gene expression decreased in BAA-1556 (0.387-fold change, (*mecA* gene-expression level after ICG-PDT)/(expression level prior to ICG-PDT), ***p < 0.001, Bonferroni *t* method). There were no significant changes in MRSA/JD39452 (*p* > 0.05).

### 3.6. Reduction of PBP2a by ICG-PDT

**Figure 5:**
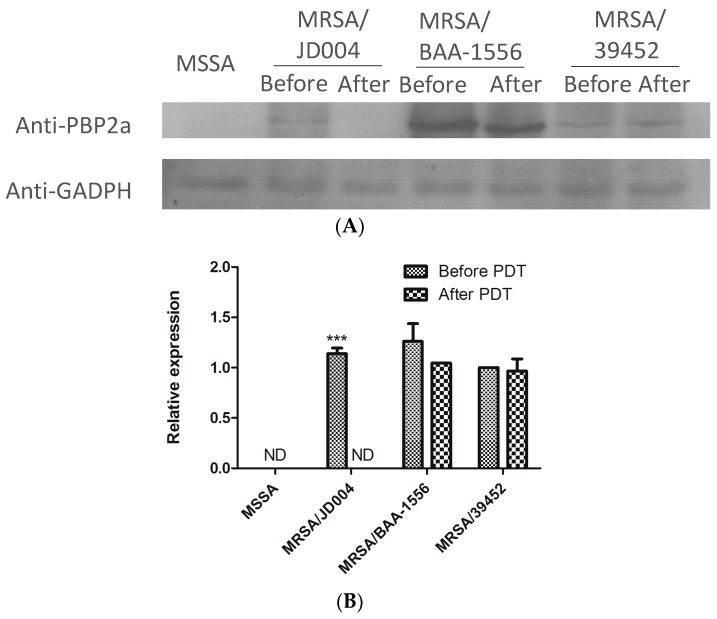
PBP2a protein expression in three MRSA strains after ICG-PDT. (**A**) After treatment, no protein expression was detected in MRSA/JD004. (**B**) 16.9% reduction in protein expression was detected in MRSA/BAA-1556, and 3.5% reduction in MRSA/39452. Each bar is the mean of two determinations ± standard deviation. Data were based on the average of three separate experiments, *** *p* < 0.001, paired Student *t*-test (PBP2a expression level after ICG-PDT/expression level prior to ICG-PDT).

PBP2a protein reduction after ICG-PDT provided additional evidence of drug-sensitivity alteration. Parallel to gene expressions, PBP2a was undetectable in MRSA/JD004 after ICG-PDT (*** *p* < 0.001, paired Student *t*-test, Figure 5). Treatment also reduced PBP2 expression in BAA-1556 and MRSA/JD39452 by 16.9% and 3.5%, respectively (not significant, *p* > 0.05, paired Student *t*-test, Figure 5).

### 3.7. Oxacillin Decreased Skin Necrosis in Mice Infected with MRSA post-ICG-PDT

**Figure 6:**
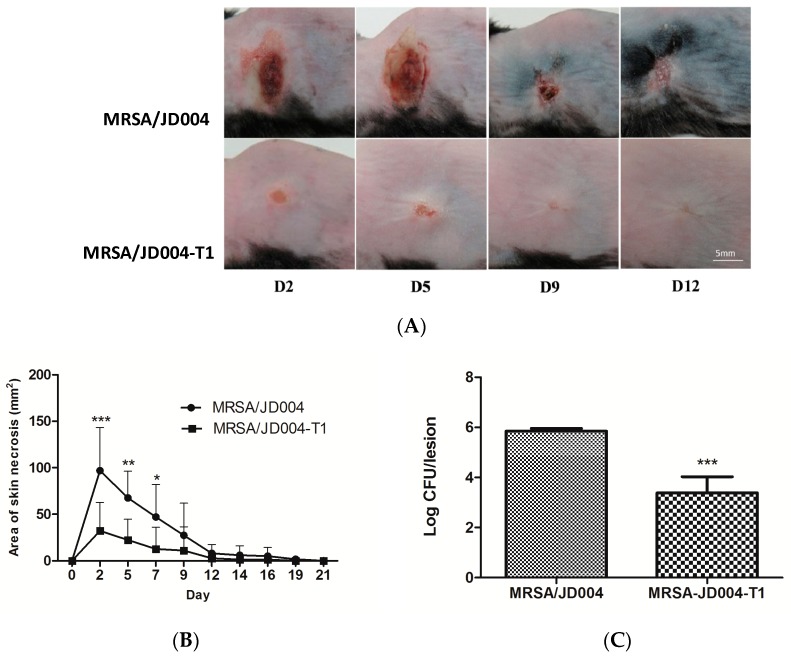
Extent of skin necrosis from mice subcutaneously inoculated with MRSA/JD004 and MRSA/JD004-T1. (**A**) Marked necrosis in mouse skin inoculated with MRSA/JD004 compared to that inoculated with MRSA/JD004-T1. Skin completely healed a week earlier in the MRSA/JD004-T1 group. (**B**) Area of skin necrosis. The data are means ± the standard deviations of the mean (*n* = 6 in each group, *** *p* < 0.001, ** *p* < 0.01, * *p* < 0.05, Bonferroni *t*-method). (**C**) Bacterial density in skin lesions three days after inoculation (*n* = 5 in each group, *** *p* = 0.0008, paired *t*-test).

To determine whether ICG-PDT-induced drug sensitivity in MRSA maintained its phenotype in vivo, mouse-skin samples were infected with MRSA/JD004-T1 and their parental cells, followed by oxacillin treatments. Mouse skin inoculated with MRSA/JD004 or MRSA/JD004-T1 developed skin necrosis that was maximized on Day 2 after inoculation and decreased with time (Figure 6). The mean areas of skin necrosis significantly differed between two groups (*** *p* < 0.001, Bonferroni *t*-method, Figure 6B). In both groups (*n* = 6 in each group), all mice developed skin necrosis, but the mean areas of necrosis were smaller in the MRSA/JD004-T1 group. Skin necrosis in this group healed around a week earlier than in mice infected with parental bacteria (*** *p* < 0.001 on Day 2, ** *p* < 0.01 on Day 5, and * *p* < 0.05 on Day 7, Bonferroni *t*-method, Figure 6B). The number of bacteria in the skin lesions infected with MRSA/JD004-T1 was significantly lower than in those infected with MRSA/JD004 (*** *p* = 0.0008, paired *t*-test, Figure 6C) three days after bacterial inoculation.

### 3.8. Oxacillin Decreased Pulmonary Bacterial Burden Following Infection with ICG-PDT/PTT-Treated MRSA

**Figure 7:**
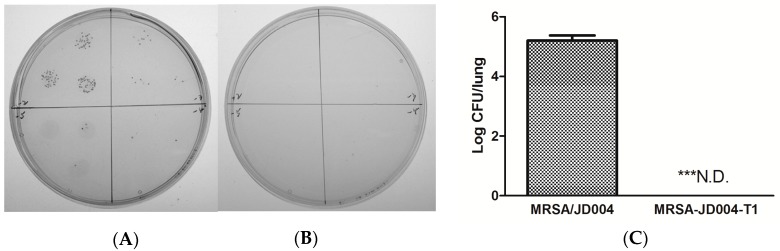
In vivo bacterial counts after lung infections with MRSA-L by nose inoculation of bacteria. Pneumonia was established in C75BL/6 mice (*n* = 5 in each group) by inoculating with (**A**) MRSA/JD004 or (**B**) MRSA/JD004-T1 through the nose. Three doses of 400 mg/kg oxacillin were intraperitoneally injected after infection. No bacteria could be recovered from lung homogenized fluid from mice infected with MRSA/JD004-T1. (**C**) Statistical data pooled from all mice. N.D.: none detectable, *** *p* = 0.0004, paired Student *t*-test.

To further investigate the systemic infection by ICG-PDT-treated MRSA, an experimental lung infection was performed. Unexpectedly, no bacteria could be recovered from the lungs of mice infected with MRSA/JD004-T1 followed by three doses of oxacillin. Five logs of bacteria could be isolated from the lung infected with parental cells (MRSA/JD004) followed by the same oxacillin treatment (*n* = 5 in each group, *p* = 0.0004, paired Student *t*-test, Figure 7).

## 4. Discussion

To the best of our knowledge, this is the first time in the literature that we have found that ICG-PDT drives MRSA toward a drug-sensitive phenotype. Photodynamic inactivation of the bacteria was positively related to drug sensitivity and was independent of *mec* complex typing.

According to Kondo et al., the *mec* typing of MRSA/JD004 belonged to class A and BAA-1556 belonged to class B. In Taiwan, some *mecA*-positive *S. aureus* may be sensitive to oxacillin [43]. The MIC test in the present study confirmed that all three strains are resistant to tested antibiotics before ICG-PDT treatment. MRSA/JD004 was the most sensitive strain to ICG-PDT (1% survived after ICG-PDT), while MRSA/JD39452 was the most resistant to ICG-PDT/PTT (32% survived after ICG-PDT). These results suggest that ICG-PDT inactivation of MRSA might be independent of the *mec* complex typing. The *mecR1* and *mecI* genes were deleted in MRSA/JD004, while *IS431* remained unaffected after ICG-PDT, suggesting ICG-PDT treatment might cause gene deletions in a novel way that does not involve *IS431*. Recently, the in vitro synergistic effect of ICG-PDT and oxacillin on MRSA clinical isolates was reported by Iluz et al. [44]. In their report, MRSA regained drug resistance in the absence of PDT. The authors explained that porphyrin-mediated PDT might damage the bacterial cell wall and cell membrane without DNA injury. Our results show that novel and persistent gene mutation or deletion occurred after ICG-PDT, and the phenotype persisted for at least 10 passages without PDT. Multiple types of cellular-component damage in a bacterium have been shown in our system [26]. This difference may be explained by the different photosensitizer we used. We believe the selection of a specific photosensitizer is critical to the success of aPDT in MRSA.

Drug-resistant *S. aureus* requires more glucose molecules to incorporate into the cell-wall peptidoglycan, which can lead to a heavy burden on ATP supply and essential key metabolites for cell replication [40]. Ender et al. showed that the transformation of a Type I *SCCmec* element into *S. aureus* yielded highly oxacillin-resistant transformants with a reduced growth rate compared to the susceptible parent. Growth rate was regained when the *SCCmec* gene was excised [41]. Our results showed a positive correlation between photodynamic inactivation and MRSA growth rates. The most susceptible strain to PDT (99% inactivation), MRSA/JD004, grew faster after PDT in a manner similar to the MSSA strains. We used single colonies in our experiments, so the possibility of MSSA contamination was minimized. Disk diffusion and MIC results further confirmed resistant phenotypes in our experiments.

Cell-wall thickening is associated with adaptive resistance to amikacin in MRSA clinical isolates and in an in vitro-induced strain [42]. Cui et al. showed that cell-wall thickening is a common feature with vancomycin resistance in *S. aureus* [40]. Vancomycin-resistant strains had considerably thickened cell walls, which became thinner with the loss of vancomycin resistance during passages without drug stimulations. Cell-wall thickness recovered when the bacteria regained a resistant phenotype. Our data support the theory that cell-wall thickness is associated with methicillin resistance in *S aureus.*

According to the CLSI guidelines, MRSA drug sensitivity was examined with oxacillin and cefoxitin instead of methicillin [45]. Methicillin is unstable and is no longer commercially available. However, the gold standard for identifying MRSA is to detect the *mecA* gene, or its product, PBP2a. Cefoxitin is a good inducer of the *mecA* gene, and cefoxitin tests give more reproducible and accurate results than oxacillin tests [46]. Our results showed a positive correlation between ICG-PDT-induced growth inhibition and a trend toward a drug-sensitive phenotype (Figure 2). It is well known that improper antibiotic dosage can induce adaptive resistance [42]. Interestingly, repeatedly treating MRSA with ICG-PDT up to eight times did not induce adaptive resistance in MRSA/JD39452, the most insensitive strain to ICG-PDT (Figure 4D). In addition, the acquired drug sensitivities in erythromycin, a 50S ribosomal subunit-targeting antibiotic, suggested that ICG-PDT had damaged multiple organelles in the bacterium and might have reduced multiple drug resistance. It also suggested that ICG-PDT does not induce drug resistance in survived cells. On the other hand, ICG-PDT might selectively damage MRSA rather than MSSA in a mixed population of wound isolates because resistant cells are more sensitive to ICG-PDT [47].

Oxacillin sensitivity in MRSA after ICG-PDT was further confirmed in the skin- and lung-infection model in mice. The significant reduction of bacterial amount in the skin after a single dose of oxacillin, and the failure to isolate bacteria from lung tissue infected with MRSA/JD004-T1 after three doses of oxacillin in the experimental pneumonia model, are robust examples of the alteration of drug sensitivity by ICG-PDT in vivo.

## 5. Conclusions

The study demonstrated that ICG-PDT drives the drug-resistant *S. aureus* toward a sensitive phenotype at least partly through *mecA* deletion and cell-wall alteration. ICG-PDT may have the potential to become an adjuvant or alternative therapy of MRSA infection.

## Supplementary Materials

The following are available online at https://www.mdpi.com/2077-0383/8/3/411/s1, Figure S1: Indocyanine green (ICG)-photodynamic inactivation (25 µg/mL ICG, near-infrared (NIR) 100 J/cm^2^ at 65.5 mW/cm^2^) of three clinical methicillin-resistant *Staphylococcus aureus* (MRSA) strains evaluated with disk-diffusion assay. Bacteria counts in CFU/mL of (A) MRSA/JD004, (B) MRSA/BAA-1556, and (C) MRSA/39452 were calculated using serial dilution and bacterial drop-plate count methods. An agar plate was allocated into four quadrants. Each quadrant was reserved for one dilution in the 10-fold serial dilutions. Each quadrant contained three drops of 20 µL bacterial suspension. (D) Average data were pooled from three independent experiments with triplicated samples. ** *p* < 0.01, and *** *p* < 0.001 in comparison with the absolute control group. Each bar was the mean of three determinations ± standard deviation. C: absolute control; DC: dark control; LC: light control; PDT: photodynamic therapy. Figure S2: *Mec* complex typing of three MRSA clinical isolates. MRSA/JD004, MRSA/BAA-1556, and MRSA/39452 were isolated from patients’ wounds. *Mec* genes were typed according to Kondo et al., using the multiplex PCR method. MRSA/JD004 and MRSA/39452 belonged to class A and MRSA/BAA-1556 belonged to class B. Table S1: Breakpoints of MRSA inhibition-zone diameters and minimum-inhibitory-concentration (MIC) values according to the CLSI criteria; Table S2: The PCR primers used in this study.
