# Supplementary material for: Indocyanine Green—Mediated Photodynamic Therapy Reduces Methicillin-Resistant Staphylococcus aureus Drug Resistance
Source: J Clin Med. 2019 Mar 25;8(3):411. doi: 10.3390/jcm8030411 (PMC6463108; doi:10.3390/jcm8030411)
Supplement: Supplementary file 1 [file jcm-08-00411-s001.pdf]

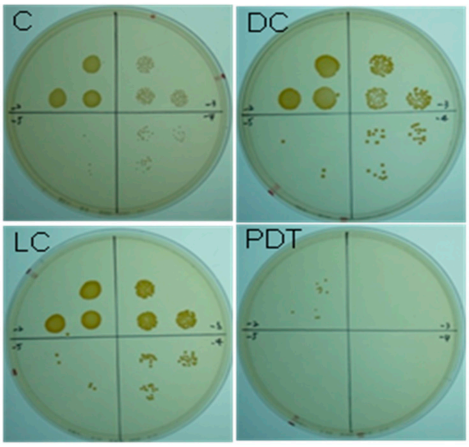

(A)

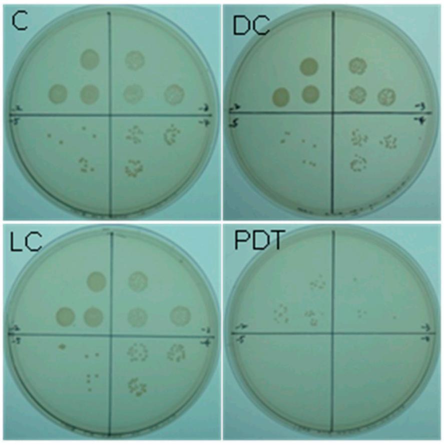

(B)

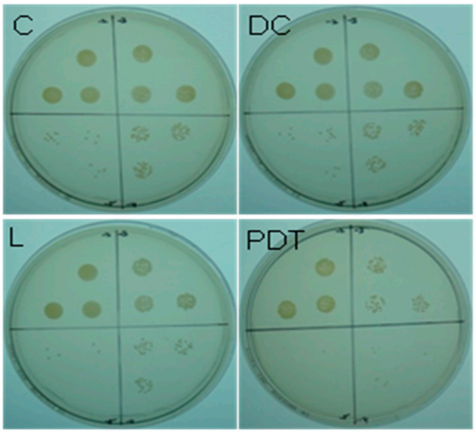

(C)

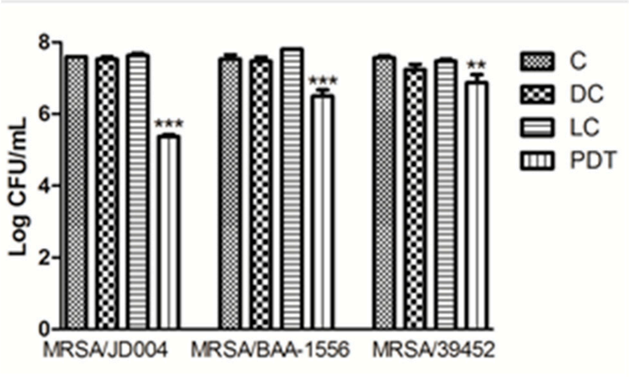

(D)

Figure S1. Indocyanine green (ICG)-photodynamic inactivation (25 μg/mL ICG, near-infrared (NIR) 100 J/cm^2 at 65.5 mW/cm^2) of three clinical Methicillin-resistant *Staphylococcus aureus* (MRSA) strains evaluated with disk-diffusion assay. Bacteria counts in CFU/mL of (**A**) MRSA/JD004, (**B**) MRSA/BAA-1556, and (**C**) MRSA/39452 were calculated by serial dilution and bacterial drop-plate count methods. An agar plate was allocated into four quadrants. Each quadrant was reserved for one dilution in the 10-fold serial dilutions. Each quadrant contained three drops of 20 μL bacterial suspension. **(D)** Average data were pooled from three independent experiments with triplicated samples. ** $p < 0.01$, and *** $p < 0.001$ in comparison with the absolute control group. Each bar was the mean of three determinations ± standard deviation. C: absolute control; DC: dark control; LC: light control; PDT: photodynamic therapy.

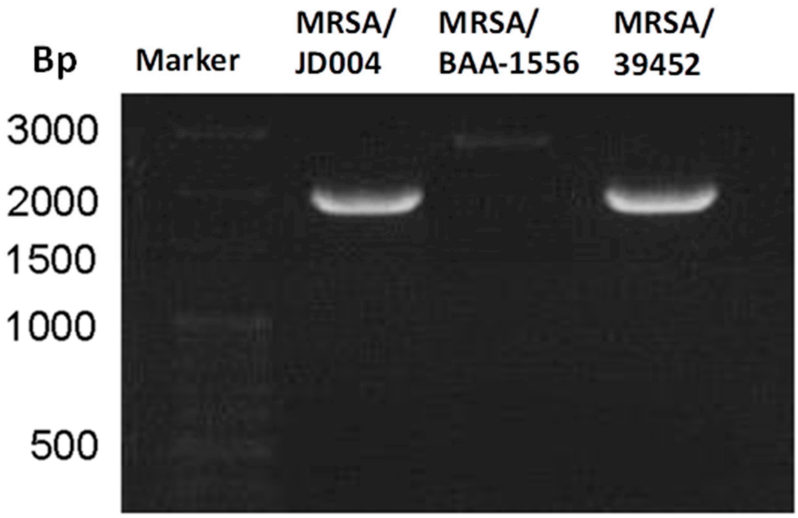

Figure S2. *Mec* complex typing of three MRSA clinical isolates. MRSA/JD004, MRSA/BAA-1556, and MRSA/39452 were isolated from patients' wounds. *Mec* genes were typed according to Kondo et al., using the multiplex PCR method. MRSA/JD004 and MRSA/39452 belonged to Class A and MRSA/BAA-1556 belonged to Class B.

Table 1. Breakpoints of MRSA inhibition-zone diameters and minimum-inhibitory-concentration (MIC) values according to the CLSI criteria.

Antibiotics	Disk content	Zone diameter (mm)		MIC value (μg/ml)	
		Resistant	Sensitive	Resistant	Sensitive
**Oxacillin (OX)**	1 μg	≦10	≧13	≥4	≤2
Cefoxitin (FOX)	30 μg	≦21	≧22	≥8	≤4

Table 2. The PCR primers used in this study.

Genes	Primers	Amplification size (bp)	References
mecA	**5' ATGAAAAAGATAAAAATTGTTCCAC 3′**<br>**5' TTATTCATCTATATCGTATTTTTTATT 3′**	**2007**	
mecR1	**5' TGGTATTTGGTTTAGTGAA 3′**<br>**5' GATTAGGTTTAGGCATTGA 3′**	**414**	**[46]**
mecI	**5' AATGGCGAAAAAGCACAACA 3′**<br>**5' GACTTGATTGTTTCCTCTGTT 3′**	**480**	**[46]**
IS1272	**5'- GTGATTCTATATGCCTACACACAA TC -3'**<br>**5'- AGCAATAAAAAAATAAATCATAATGAGTCC - 3'**	**1512**	

Genes	Primers	Amplification size (bp)	References
IS431	**5'-ATGAACTATT TCAGATATAAACAATTTAACA -3'**<br>**5'- TTAACTTGCTAGCATGATGCTA -3'**	**674**	
16s rRNA	**5'-GTTATTAGGGAAGAACATATGTG-3'**<br>**5'-CCACCTTCCTCCGGTTTGTCACC-3'**	**750**	**[47]**
